# Catheter ablation in a monochorionic diamniotic twin pregnancy: A case report and literature review

**DOI:** 10.1097/MD.0000000000040443

**Published:** 2024-11-01

**Authors:** Yanxi Jia, Hua Liao, Qing Hu, Hongyan Liu, Zhaomin Zeng, Haiyan Yu

**Affiliations:** a Department of Obstetrics and Gynecology, West China Second University Hospital, Sichuan University, Chengdu, China; b Key Laboratory of Birth Defects and Related Diseases of Women and Children (Sichuan University), Ministry of Education, Chengdu, China.

**Keywords:** arrhythmia, preexcitation syndrome, radio-frequency ablation, twin pregnancy

## Abstract

**Rationale:**

Preexcitation syndrome is an uncommon congenital cardiac disorder that impairs the normal cardiac conduction system. Radiofrequency ablation is one of the most effective treatments for this condition. Nevertheless, radiofrequency ablation is rare in women with preexcitation syndrome during pregnancy.

**Patient concerns:**

A 33-year-old woman with monochorionic diamniotic twin pregnancy complicated by sinus arrhythmia with ventricular preexcitation at 14 weeks and 5 days of gestation, with paroxysmal palpitations and shortness of breath at 16 weeks with paroxysmal supraventricular tachycardia with preexcitation syndrome and a heart rate ranging from 180 to 225 beats per minute.

**Diagnoses:**

The pregnant occurred sudden palpitations and shortness of breath in the shower. Electrocardiography revealed paroxysmal supraventricular tachycardia, and electrophysiological study revealed preexcitation syndrome (dominant accessory route of the left free wall) with atrioventricular reentrant tachycardia.

**Interventions:**

Radiofrequency catheter ablation was performed at 20 weeks.

**Outcomes:**

Symptoms of preexcitation syndrome resolved after the radiofrequency catheter ablation, and 2 healthy infants were delivered at 36 weeks and 2 days of gestation by cesarean section.

**Lessons:**

Preexcitation syndrome may result in life-threatening arrhythmias such as supraventricular tachycardia during pregnancy and delivery. It might be efficiently controlled through optimal treatment by a multidisciplinary team, which would effectively minimize arrhythmia risk events during pregnancy and improve maternal–fetal outcomes. Based on the patient’s individual situation, radiofrequency ablation may be a procedure in pregnant women with preexcitation syndrome.

## 1. Introduction

Preexcitation syndrome, a congenital disorder concerning the Kent bundle that affects the normal cardiac conduction system (CCS), is characterized by one or more accessory pathways (AP) that provide direct cohesiveness between the atrial and ventricular myocardium, forming a route for reentrant tachycardia circuits.^[1,2]^ It can be divided into the following 3 parts: Wolf–Parkinson–White syndrome (WPW), Lown–Ganong–Levine syndrome, and Mahaim-Type preexcitation.^[3]^

A survey indicated that the prevalence of preexcitation syndrome is 0.1% to 0.3% in the general population. However, over 65% of teenagers and 40% of adults present no clear clinical manifestations, leading to the neglect of timely management.^[4]^ A guideline states that paroxysmal supraventricular tachycardia is the most prevalent arrhythmia in patients with preexcitation syndrome, followed by atrial fibrillation.^[1]^ Pregnant women are more likely to develop cardiovascular diseases, and arrhythmia is a major concern.

The WPW syndrome, as the most prevalent type of preexcitation syndrome, is characterized by a short PR interval (≤120 ms) and a prolonged QRS duration (>120 ms), which, in the presence of sinus rhythm, inscribes a “delta” wave that causes the QRS complex to initially slur upstroke or downstroke.^[5]^ Nevertheless, the location, type of conduction, and whether they can conduct retrogradely, anterogradely, or in both directions constitute some of the features that may be utilized to define WPW syndrome. Normally, based on the electrocardiography (ECG) findings and clinical presentation, it can be classified as follows:

1. Type A: “delta” wave, prolonged QRS complex, and dominant ST segment (negative), with the bypass occurring around the left atrioventricular annulus.
2. Type B: “delta” wave, prolonged QRS complex and dominant ST segment (positive), with the bypass occurring around the right atrioventricular annulus.
3. Type C: only prolonged QRS complex, ST segment was dominant (negative).

According to certain studies, the left atrioventricular annulus accounted for 58% of the APs.^[6]^ Since the atrioventricular node is bypassed by an auxiliary route, syncope, and palpitations are common symptoms of WPW, and other manifestations include episodic lightheadedness, presyncope, or even cardiac arrest.^[7]^ The treatment options for this condition may include medication, cardioversion, and radiofrequency catheter ablation.

Herein, we report a case of monochorionic diamniotic twin pregnancy with preexcitation syndrome (left free-wall dominant bypass) and successful radiofrequency catheter ablation (RFCA) during pregnancy. Additionally, we used a list of keywords, including preexcitation, radiofrequency ablation, and pregnancy to conduct a literature review in English and Chinese. The study was approved by the Ethics Committee of West China Second University Hospital.

## 2. Case presentation

**Figure 1.**
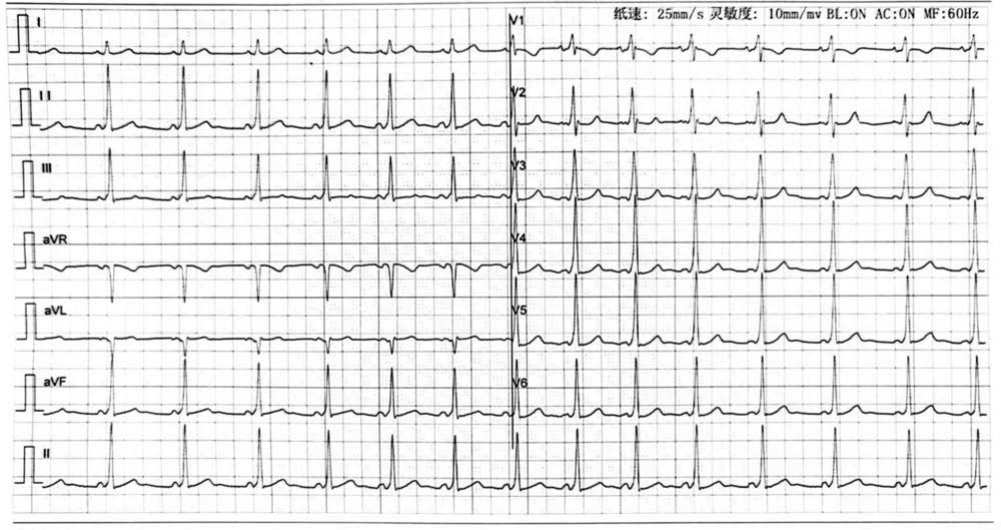
ECG revealed sinus arrhythmia with ventricular preexcitation at the gestational age of 14 weeks and 5 days. ECG = electrocardiography.

A 33-year-old woman (gravida 1, para 0) underwent in vitro fertilization and embryo transfer. At 12 weeks and 5 days of gestation, ultrasound examination revealed a monochorionic diamniotic twin pregnancy. ECG at 14 weeks and 5 days of gestation showed sinus arrhythmia with ventricular preexcitation (Fig. 1), and the echocardiography was normal. The pregnant woman did not show any signs of abnormal cardiac rhythm or physical discomfort, and had no history of medication, allergies, inherited disorders, use of prescription drugs, substance abuse, and family history of arrhythmia and congenital heart diseases.

**Figure 2.**
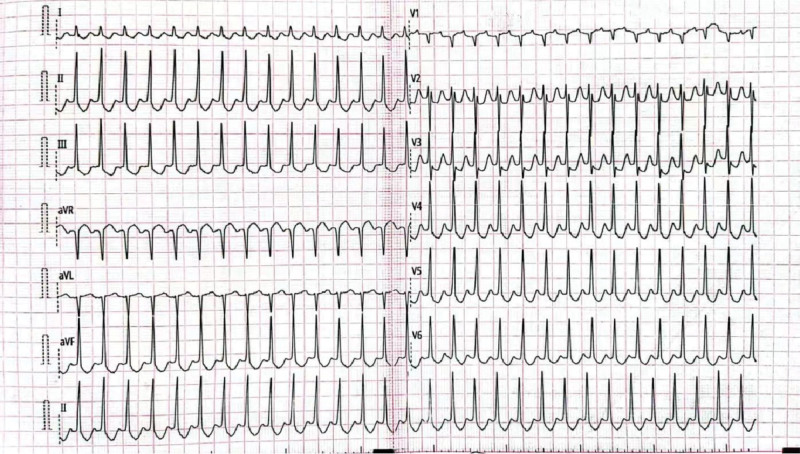
ECG revealed paroxysmal supraventricular tachycardia (PSVT) at 16 weeks of gestation. ECG = electrocardiography.

At 16 weeks of gestation, the pregnant occurred sudden palpitations and shortness of breath in the shower, and was transferred to the hospital. Her heart rate ranged from 180 to 225 beats per minute (bpm). Serum electrolytes showed the potassium concentration was 3.2 mmol/L. While the results were normal in routine blood examination, liver and renal function, and myocardial markers. ECG (Fig. 2) revealed “PSVT,” echocardiography revealed no obvious abnormality, with regard to the particularity of the woman, she was evaluated by a cardiologist. Then vagal maneuvers was tried, intravenous adenosine triphosphate and oral potassium supplementation were performed. The patient recovered sinus rhythm with a heart rate of 80 to 95 bpm following the aforementioned procedures.

**Figure 3.**
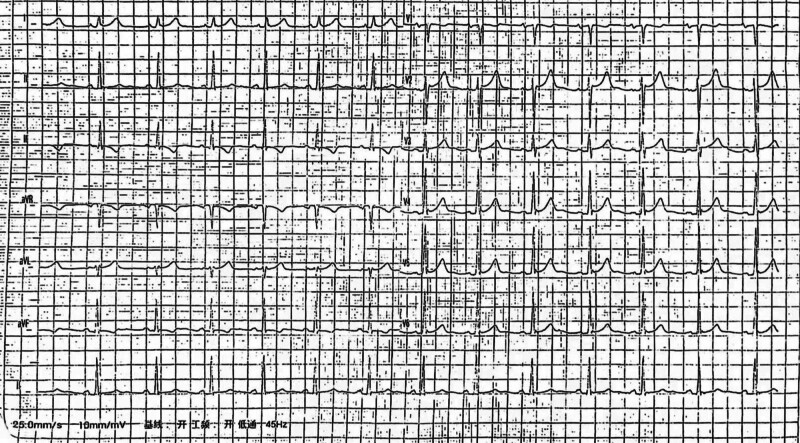
ECG at 1 day after RFCA. ECG = electrocardiography, RFCA = radiofrequency catheter ablation.

Close monitoring was then performed to the mother and the fetuses. Due to her recurrent palpitation and the couple’s willing to continue twin pregnancy, the potential hazards of long-term medication and the increased risks of ventricular fibrillation in twin pregnancy were considered seriously. Thus, the pregnant opted for RFCA at 20 gestational weeks after extensively counseled by obstetrician and cardiologist. The electrophysiological study was carried out under the direction of a three-dimensional (3D) navigation system (CARTO, Biosense Webster Inc., Diamond Bar, CA) during the procedure, which revealed preexcitation syndrome (dominant accessory route of the left free wall) with atrioventricular reentrant tachycardia. Ultimately, the procedure was successful and the pregnant had no palpitations, chest tightness, chest pain and with normal ECG (Fig. 3) after the operation.

**Figure 4.**
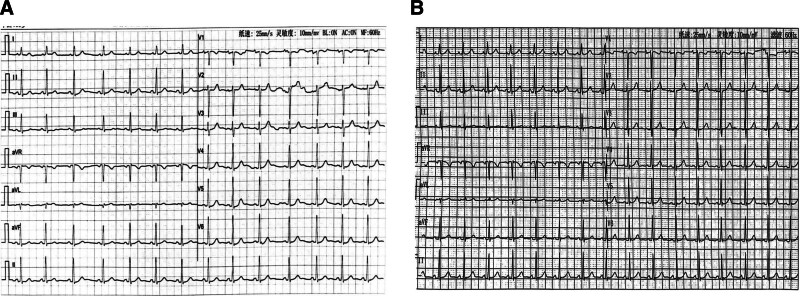
(A) ECG at gestational age of 24 weeks and 6 days. (B) ECG at gestational age of 33 weeks and 6 days. ECG = electrocardiography.

The woman and the fetus were closely followed up. The pregnancy was uneventful, and the woman remained asymptomatic with normal ECG (Fig. 4) and echocardiography. Based on the evaluation by a multidisciplinary team (MDT), anesthesiologists, obstetricians, cardiologists, neonatologists, 2 babies were delivered by elective cesarean section at 36 weeks and 2 days of gestation, the first baby weighed 2460 g with Apgar scores of 10 and 10 at 1 and 5 minutes, and the second baby weighed 2410 g with the same Apgar scores of the first baby.

**Figure 5.**
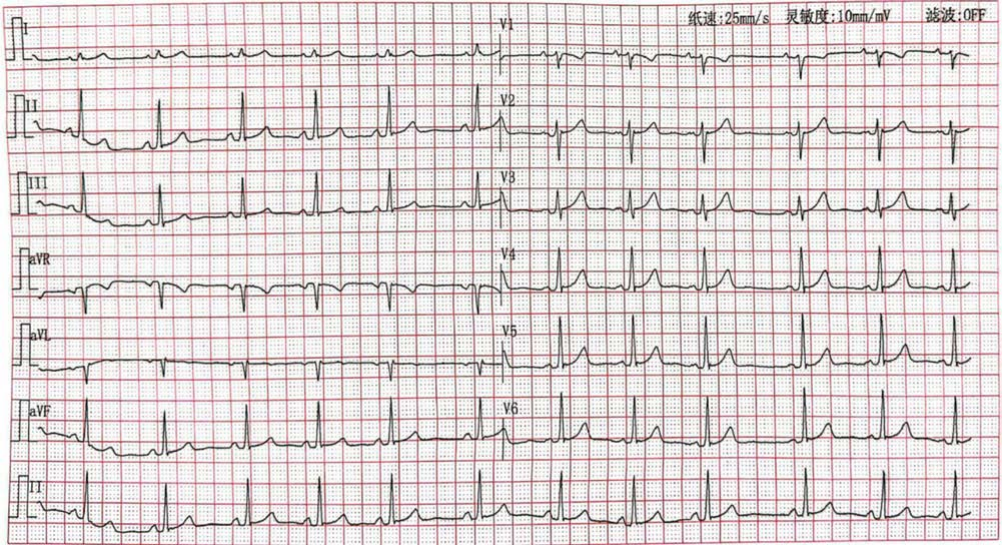
ECG at 9 months after RFCA. ECG = electrocardiography, RFCA = radiofrequency catheter ablation.

Until now, the mother and twins were all in good health condition during 6 months follow-up. The woman had no palpitations or syncope, and ECG (Fig. 5) was still normal.

## 3. Discussion

Preexcitation syndrome is an umbrella term for the existence of additional congenital atrioventricular channels or bypass pathways in addition to the normal atrioventricular conduction system, and frequently results in arrhythmias.

A recent nationwide assessment in the United States found that the incidence of arrhythmia during pregnancy is 139/100,000 and has been on the rise. Nevertheless, since arrhythmias are not examined independently from other cardiovascular etiologies in the Pregnancy Mortality Surveillance System, it is unknown how precisely arrhythmias contribute to this increased mortality. Clinical symptoms of arrhythmia, such as palpitations, exhaustion, dizziness, chest pain, dyspnea, and altered awareness, may develop during pregnancy or worsen. The extent to which hemodynamics are affected by arrhythmia determines the severity of the symptoms.^[8]^

The co-occurrence of tachyarrhythmias normally reoccurs with a manifest AP that results in the so-called preexcitation syndrome, referred to as “WPW syndrome.” A short PR interval (0.12 seconds) and a delta wave with an extended (0.12 seconds) QRS complex are features of WPW syndrome on the ECG, while the Lown–Ganong–Levine syndrome is characterized by a short PR interval, normal QRS complex, and paroxysmal tachycardia.^[9]^ According to several studies, surface ECG algorithms have been designed to determine the localization of APs in the presence of overt preexcitation; nevertheless, further clinical research is required to confirm the practicality and efficiency of these algorithms.^[10,11]^ The role of WPW therapy during pregnancy remains controversial.

Pregnancy has been associated with an increased incidence of cardiac arrhythmias and an increased risk of supraventricular tachycardia (SVT), particularly in patients with preexcitation syndromes. Although the precise mechanism responsible for these elevated risks remains unknown, it could be associated with the synergistic effects of autonomic, hormonal, and hemodynamic alterations. For one thing, the effective circulation blood volume increases by 30% to 50% beginning at week 8 and peaks between weeks 32 and 34 of gestation. Here, expanding atrioventricular cells activate stretch-activated ion channels, resulting in early afterdepolarization, shorter refractory period, slower conduction, and greater spatial dispersion.^[12]^ For another, an individual may experience arrhythmia due to changes in autonomic and hormonal processes, as pregnancy increases the susceptibility to adrenergic stimuli, such as stress, anxiety, or estrogen. Specifically, estrogen is hypothesized to enhance the quantity and sensitivity of alpha-adrenergic receptors, adrenergic receptors in the hypothalamus, and excitability of uterine muscle fibers. This combination has the potential to induce arrhythmias by changing the refractory period or conduction velocity in the reentrant circuit.^[13]^ In this case, the woman was characterized by twin pregnancy, who carried a higher cardiac stress and risk of arrhythmia.

When it comes to treating preexcitation syndrome, asymptomatic adults are generally left untreated; instead, they should be monitored closely for any abnormalities in their ECG, and approximately one in 5 of these patients will develop an arrhythmia related to their AP during follow-up.^[1]^ Nevertheless, unlike patients with silent arrhythmias, especially during pregnancy, those with symptoms should have personalized treatment plans that take into account their unique clinical presentation and ECG expression. Therefore, given the characteristics of preexcitation syndrome and the importance of therapy, the case reported is a candidate for more intensive therapies.

Preexcitation syndrome during pregnancy frequently gets treated with a combination of medicinal therapy and RFCA. There are no specific recommendations for the use of anti-arrhythmic medications during pregnancy. Depending on the type of arrhythmia and underlying cardiac conditions, the urgency of the indication will determine whether a pregnant woman with arrhythmia needs medicinal therapy.

The European Society of Cardiology guidelines^[1]^ state that before becoming pregnant, patients with a history of ventricular tachycardia or SVT may recommended to consider RFCA. When vagal stimulation or adenosine is ineffective or impractical for treating SVT during pregnancy with preexcitation syndrome, RFCA is a class 1 suggestion for hemodynamically stable patients. Nevertheless, synchronous cardioversion (Class 1) is recommended whenever the patient is unstable during acute therapy. Furthermore, it is advisable to refrain from using antiarrhythmic drugs during the first trimester of pregnancy when it comes to the timing of taking medications during pregnancy. According to the guideline, RFCA should be performed in an experienced center, using catheter navigation devices and non-fluoroscopic electroanatomical mapping.^[14]^

As is well known, arrhythmia has a high recurrence throughout pregnancy, which raises the risk of adverse events for both the mother and the fetus, and it can considerably increase the chance of maternal death. Pregnancy complicated by tachyarrhythmia, which may first appear or continue to worsen during pregnancy, can lead to certain complications such as maternal heart failure and abrupt death, fetal growth restriction, fetal distress, preterm birth, still birth, and other negative outcomes. Thus, close monitoring and timely intervention ought to be taken into serious consideration to prevent severe maternal and perinatal consequences. Additionally, the mother’s lower cardiac output, which also causes a decrease in uterine blood flow, places the fetus in a state of relative hypoxia. The above can often lead to fetal distress, still birth, fetal growth restriction, preterm birth, and low birth weight infant, etc. In other words, provided that adequate and effective treatment is neglected, the fetus and pregnant women will be at risk.

Therefore, in this instance, we chose to perform RFCA and electrophysiological study followed successful intravenous adenosine triphosphate under the supervision of MDT due to the pregnant woman’s twin pregnancy following embryo transfer, the higher cardiac load and the higher rate of ventricular fibrillation caused by SVT than in other groups, and the potential harm that long-term antiarrhythmic medication utilization may cause to the developing fetus. And this unique treatment has been proven to be successful in this case. Furthermore, this was in line with earlier reports’ recommended course of action and instances in which RFCA was carried out.^[1,8,15–18]^ A retrospective analysis of 309 pregnancies in 280 women with preexcitation syndrome who delivered at West China Second University Hospital from June 2011 to October 2021 revealed that 5 of 29 women, who had 2 pregnancies in ten years, had received RFCA after the first delivery, and none of them had SVT in the second pregnancy or delivery.^[19]^

Accordingly, throughout this case, we conclude that the choice of treatment for patients with preexcitation syndrome during pregnancy should be based on the characteristics of the patients as well as taking into account the application recommendation level of treatment methods specified in the published guidelines. Moreover, we summarized the clinical presentations and features of our case and reported preexcitation syndrome during pregnancy performed RFCA (Table 1).^[20–26]^ The first was reported in 1999,^[20]^ which involved a 31-year-old woman who had frequent episodes of SVT associated with exercise and sometimes with syncope since the age of 12 years, and suffered preexcitation during her gestational age of 20 weeks, but then got well and had superior pregnancy outcomes at the postpartum follow-up after RFCA. In earlier instances, we can conclude that the presentations of preexcitation syndrome during pregnancy can practically be palpitations, chest discomfort, and dyspnea without any evident causes in the process of pregnancy, and this syndrome mostly occurs in the second trimester (66.7%). The table results make it clear that all pregnant women were treated with RFCA following a diagnosis of preexcitation syndrome and that the majority of pregnancy terminations involved elective cesarean sections. However, as a matter of fact that the majority of previous case reports omitted information regarding the timing and methods of pregnancy termination, which may have biased the results. Hence, future research should concentrate more on determining whether variations in the timing and methods of pregnancy termination following RFCA will affect the outcomes for both mothers and fetuses. However, we should keep in mind that pregnancy complicated only by preexcitation syndrome is not an indication for delivery or cesarean section. Instead, pregnant women with preexcitation syndrome are advised to choose modes of delivery based on obstetric indications and other concerns.

**Table 1:** Summary of the clinical features of cases with preexcitation syndrome and radiofrequency ablation during pregnancy.

Study ID	Maternal age (years)	Pregnancy type	Clinical presentation	GA at ablation (wks)	GA at delivery (wks)	Delivery mode	Maternal outcome	Infant outcome
Domínguez A et al (1999)^[20]^	31	Singleton	Syncope	20	NS	NS	In good condition	In good condition
Bombelli F et al (2003)^[21]^	27	Singleton	Paroxysmal palpitations	29	41	CS	NS	The infant with retardation in cranial growth and microcephaly at 4 months after birth
Kanjwal Y et al (2005)^[22]^	32	Singleton	Recurrent palpitations, chest pain, syncope	22	NS	NS	In good condition	NS
Berruezo A et al (2007)^[23]^	22	Singleton	Self-limited palpitations, anxiety, chest pain	12	NS	NS	In good condition	NS
Manjaly ZR et al (2011)^[24]^	33	Singleton	Life-threatening arrhythmia	15	NS	NS	In good condition	In good condition
Li MM et al (2019) (total of 28 cases)^[25]^	21–37	Singleton	Palpitations and presyncope	18	34–39	NS	In good condition	In good condition
Jeong HK et al (2020)^[26]^	36	Singleton	Palpitations, chest discomfort, dyspnea	13^+1^	NS	NS	In good condition	NS
Our case	33	Twin	Palpitations	16	36^ + 2^	CS	In good condition	Both babies in good condition

CS = cesarean section; GA = gestational age; NS = not specified; wks = weeks.

Generally speaking, the prognosis and maternal-fetal outcomes for individuals with preexcitation syndrome through RFCA are favorable, yet it is worth noting that prior studies have all featured with singleton pregnancies, while this case involved a twin pregnancy.

Another study^[25]^ provided an overview of the pregnancy outcomes of patients who had sustained tachyarrhythmia episodes without structural heart disease during pregnancy from 2015 to 2018; however, only 28 of them underwent RFCA during pregnancy. Anyway, the above findings all demonstrated that in pregnant women with tachyarrhythmia or preexcitation syndrome, zero-radiation catheter ablation during pregnancy offered favorable therapeutic advantages for both the mother and fetus. Nevertheless, it is worth noting that RFCA ought to be carried out following a thorough assessment by the departments of cardiology, anesthesiology, obstetrics, and nursing.

The significance of cardiac and maternal care throughout pregnancy, particularly in twin pregnancies, is proven in this instance. The healthy birth of the twins, RFCA during the second trimester of pregnancy, and improved pregnancy outcomes following cautious administration are the salient features of this case. We believe that the patient’s medical care plays a key role in this regard; however, there is still a dearth of knowledge regarding the factors that may impact the prognosis of this pregnancy. It is unclear what specific circumstances should be met in comparable situations to offer superior prenatal care.

Although there have been a few rare cases of preexcitation syndrome in pregnant women,^[27,28]^ the primary aspects of our case were the effective medical management and the decision made by the therapeutic team to effectively safeguard the pregnancy while closely monitoring the patient.

## 4. Conclusion

In conclusion, preexcitation syndrome may result in life-threatening arrhythmias, such as SVT during pregnancy and delivery. Especially twin pregnancy, which is prone to develop a variety of cardiac conditions due to their high cardiac burden and the increase in myocardial vascular capacity. However, it can be efficiently controlled through optimal treatment by a MDT, including anesthesiologists, obstetricians, cardiologists, electrophysiologists, maternal–fetal medicine specialists, and neonatologists, which would effectively minimize the arrhythmia risk events during pregnancy and improve maternal-fetal outcome. Based on the patient’s individual situation, RFCA may be a favorable procedure in pregnant with preexcitation syndrome.

## Acknowledgments

We are grateful to the doctors and staff involved in this work. We are also grateful to the doctors and staff of the Department of Cardiology at the West China Hospital.

## Author contributions

**Conceptualization:** Yanxi Jia, Qing Hu, Haiyan Yu.

**Data curation:** Yanxi Jia, Hua Liao, Hongyan Liu, Zhaomin Zeng.

**Formal analysis:** Yanxi Jia.

**Funding acquisition:** Haiyan Yu.

**Investigation:** Hua Liao, Qing Hu, Hongyan Liu, Zhaomin Zeng.

**Supervision:** Haiyan Yu.

**Writing – original draft:** Yanxi Jia.

**Writing – review & editing:** Haiyan Yu.
